# Efficient Iris Recognition Based on Optimal Subfeature Selection and Weighted Subregion Fusion

**DOI:** 10.1155/2014/157173

**Published:** 2014-02-10

**Authors:** Ying Chen, Yuanning Liu, Xiaodong Zhu, Fei He, Hongye Wang, Ning Deng

**Affiliations:** ^1^College of Computer Science and Technology, Jilin University, Changchun 130012, China; ^2^Key Laboratory of Symbolic Computation and Knowledge Engineering of Ministry of Education, Jilin University, Changchun 130012, China; ^3^College of Software, Nanchang Hangkong University, Nanchang 330063, China; ^4^Internet of Things Technology Institute, Nanchang Hangkong University, Nanchang 330063, China

## Abstract

In this paper, we propose three discriminative feature selection strategies and weighted subregion matching method to improve the performance of iris recognition system. Firstly, we introduce the process of feature extraction and representation based on scale invariant feature transformation (SIFT) in detail. Secondly, three strategies are described, which are orientation probability distribution function (OPDF) based strategy to delete some redundant feature keypoints, magnitude probability distribution function (MPDF) based strategy to reduce dimensionality of feature element, and compounded strategy combined OPDF and MPDF to further select optimal subfeature. Thirdly, to make matching more effective, this paper proposes a novel matching method based on weighted sub-region matching fusion. Particle swarm optimization is utilized to accelerate achieve different sub-region's weights and then weighted different subregions' matching scores to generate the final decision. The experimental results, on three public and renowned iris databases (CASIA-V3 Interval, Lamp, andMMU-V1), demonstrate that our proposed methods outperform some of the existing methods in terms of correct recognition rate, equal error rate, and computation complexity.

## 1. Introduction

Nowadays, biometric recognition has become a common and reliable way to authenticate the identity of a living person based on physiological or behavioral characteristics. Iris recognition is one of the most stable and reliable technology among biometric technologies some desirable properties such as uniqueness, stability, and noninvasiveness make iris recognition particularly suitable for highly reliable human identification.

Generally speaking, traditional feature extraction approaches and corresponding iris recognition system can be divided into five major categories roughly: phase-based approaches [1–3], zero crossing approaches [4], texture analysis based approaches [5], intensity variation analysis based approaches [6, 7], and other approaches [8–13]. Most of above-mentioned literatures need convert ring-shaped (polar coordinates) iris area to Cartesian coordinates to overcome the variations and then extract features from normalized rectangular iris pattern. However, some factors, such as changes in the eye gaze, non-uniform illumination, variations in orientation or scale may bring about iris images with different-level quality. Most of tradition feature extraction methods have two drawbacks when processing this kind of different-level image. (1) Coordination transform may lead to feature missing because ring-shaped with different length. Proença and Alexandre [14] have pointed out that polar transformation may lead to problem of aliasing. They studied the relationship between size of captured iris image and its recognition accuracy and observed that the recognition accuracy reduces considerably due to change in area of iris. (2) Most conventional methods of iris recognition are unable to achieve true rotation invariance. Rotation invariance is important for an iris recognition system since changes of head orientation and binocular vergence may cause eye rotation [15]. Scale invariant feature transformation (SIFT), firstly proposed by Lowe [16], which can effectively overcome the above-mentioned shortcomings to a certain degree. SIFT method is capable of extracting and matching points which are stable and characteristic between two images. It uses both image intensity and gradient information to characterize the neighborhood property of a given landmark. The algorithm includes scale-space extreme detection, feature localization, orientation assignment, feature descriptor, and feature matching [17]. The SIFT technique has already demonstrated its efficacy in Clinical CT images [17–19], 3D images [20, 21], Omnidirectional images [22, 23], and Brodatz texture images recognition [24], and it also has been proposed using biometric recognition system based on face [25], palmprint [26], and iris images [12, 13, 15, 27, 28]. Soyel and Demirel [25] proposed a discriminative SIFT for facial expression recognition. They adopted key-point descriptors of SIFT features to construct facial feature vectors, and Kullback Leibler divergence was used for the initial classification and weighted majority voting based classifier to generate the final decision. Mehrotra et al. [12] pointed out that the scale invariant technique is found to be suitable for annular iris images because the iris size changes caused by expansion and contraction of pupil. However, traditional SIFT also has shortcomings. Soyel and Demirel [25] pointed out that the major drawback of standard SIFT technology is that it does not consider the location of the feature, which may further cause two feature keypoints corresponding to the minimum distance that could not be related to the same image part. Belcher and Du [13] proposed the region-based SIFT approach to iris recognition; they divided annular iris area into left, right and bottom subregions and then got the best matching score for three subregions, respectively; finally averaged all three best matching scores as overall matching score. However, their simply averaged operation is unreasonable method because three subregions have own different feature, and it should be assigned corresponding weights according to subregion's intrinsic information.

In this paper, we propose an efficient iris recognition system based on optimal subfeature selection strategies and subregion fusion method. This recognition system is composed of two parts. The first part is discriminative subfeature selection based on finite-delete-sorting multistage strategy, and the second one is fusion subregion of segmented annular iris area. The goal of discriminative subfeature selection is to discard the redundant SIFT keypoint's feature; the feature selection strategies include (1) feature selection based on keypoint's orientation, (2) feature selection based on keypoint's neighborhood magnitude, and (3) compounded feature selection. The purpose of weighted subregion feature fusion is to overcome the major drawback of standard SIFT technology. First, we divide segmente iris annular area into three equally sized partitions in a nonoverlapping way. Second, weighted coefficients of subregion are obtained via training with particle swarm optimization (PSO) method. Finally, we adopt weighted subregion matching to achieve final decision.

The rest of the paper is organized as follows. We first describe the feature extraction and feature representations based on SIFT detailed in Section 2.  Section 3 introduces our three proposed discriminative subfeature selection strategies.  Section 4 mainly focuses on describing the process of subregion partition, corresponding weights assignment, and weighted subregion matching. Experimental results, comparisons with state-of-the-art methods, and discussion are represented in Section 5, respectively.  Section 6 summaries this study.

## 2. Feature Extraction and Representation

**Figure 1:**
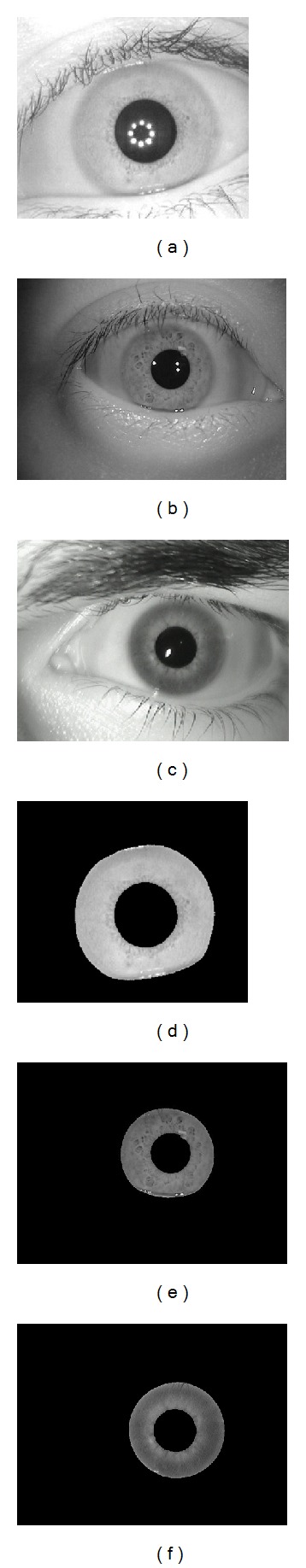
Examples of segmented iris images. (a), (b), and (c) are original iris images, (a) from CASIA-V3 Interval database (S1002L04.jpg), (b) from CASIA-V3 Lamp database (S2005R02.jpg), and (c) from MMU-V1 database (yannl5.bmp, left) database, respectively. (c), (d), and (e) are segmented iris images which correspond to (a), (b), and (c).

Before extracting iris feature, the iris image needs to be preprocessed. Locating the iris area in an iris image is an important step. In the past several years, we have done some related work on iris image preprocessing; here we adopt a coarse-to-fine segmentation method based on adaptive level set, and this localization method can segment iris area accurately and exclude eyelashes and eyelids. Meanwhile, the drawbacks of coordination transformation have been described in the introduction section. Therefore, the following extensive experiments directly consider the annular region of iris without normalization. Examples of segmented iris images are shown in Figure 1.

### 2.1. Detection of Scale-Space Extreme 

The first step is to construct a Gaussian scale space *L*(*x*, *y*, *σ*); the input image *I*(*x*, *y*) is successively smoothed with a Gaussian function *G*(*x*, *y*, *σ*) via

(1) $$\begin{array}{c} L\left(x,y,\sigma \right)=G\left(x,y,\sigma \right)*I\left(x,y\right), \end{array}$$

where *G*(*x*, *y*, *σ*) = (1/2*πσ* ^2^)*e* ^−(*x*^2^+*y*^2^)/2*σ*^2^^, and ∗ is the convolution operation in *x* and *y*.

Then the difference-of-Gaussian (DOG) images *D*(*x*, *y*, *σ*) can be computed from the difference of two nearby scales separated by a constant multiplicative factor *k* via

(2) $$\begin{array}{c} D\left(x,y,\sigma \right)=L\left(x,y,k\sigma \right)-L\left(x,y,\sigma \right). \end{array}$$

**Figure 2:**
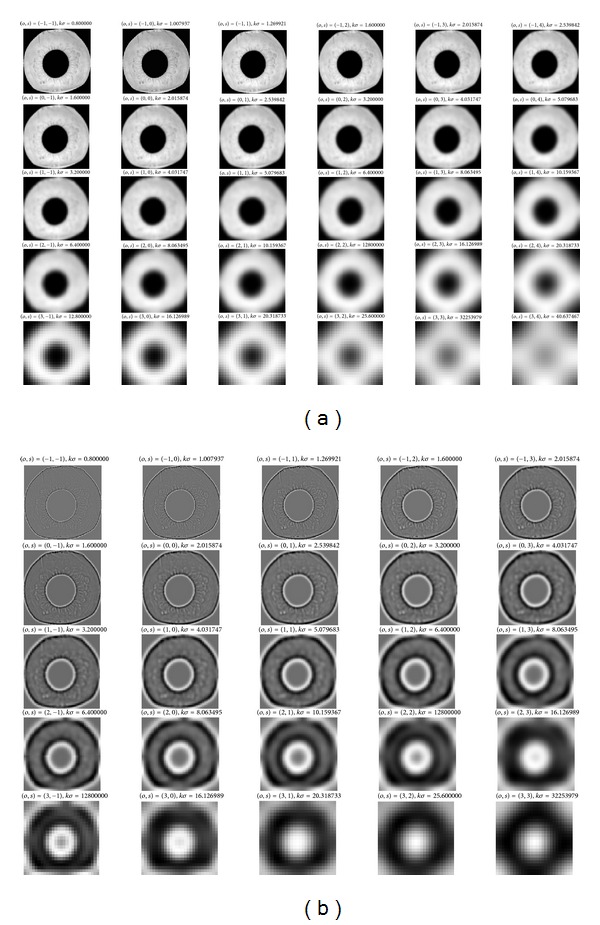
Detection of scale-space extreme. (a) Gaussian smoothed annular iris images for different octave, scale, and *σ*; (b) corresponding DOG images.

Each collection of DOG images and Gaussian-smoothed images of the same size is called an octave, and each octave of scale space is divided into an integer number *s*, so *k* = 2^1/2^. It is necessary to produce *s* + 3 images for each octave for making the final extreme detection cover a complete octave. In this paper, we set *s* = 3 scale number as 6 and octave number as 5, respectively.  Figure 2 shows the Gaussian smoothed iris images and corresponding DOG images for change in octave, scale, and *σ*.

In order to detect the local minima and maxima of DOG images *D*(*x*, *y*, *σ*), each sample point is compared to its eight neighbors in the current image and nine neighbors in the scale above and below and only sample point which is larger or smaller than all of these neighbors will be selected [29], and these minima and maxima points are called keypoints.

### 2.2. Keypoints Localization

**Figure 3:**
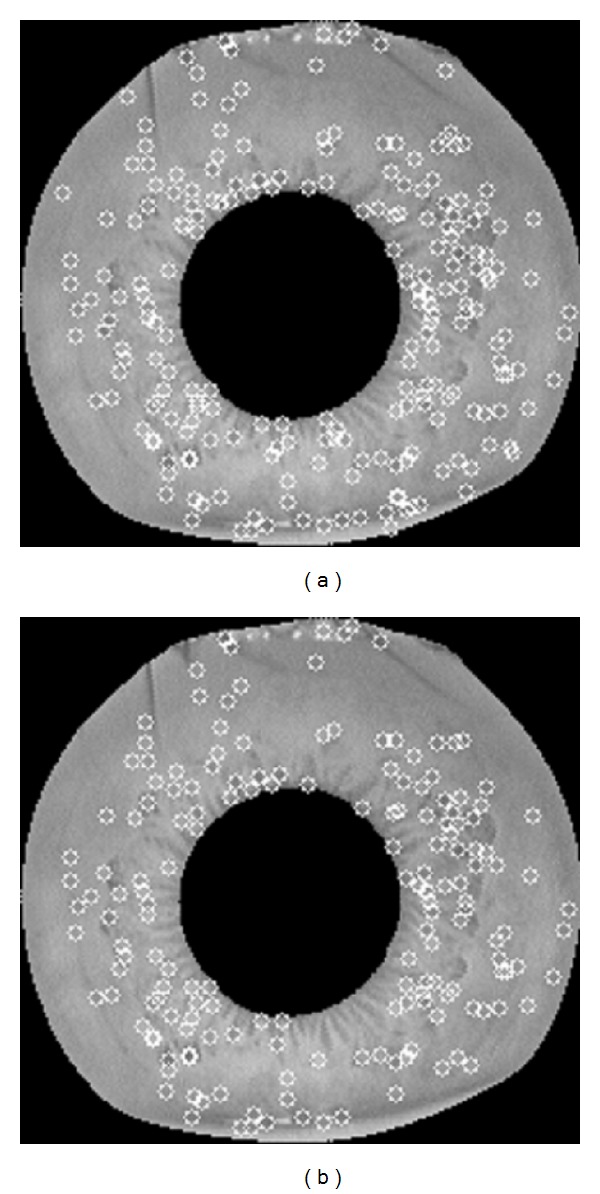
The stages of keypoints selection on annular iris image using SIFT. (a) shows the 267 keypoints at all detected maxima and minima of DOG function, and (b) shows the final 216 keypoints remaining after applying a threshold on minimum contrast (0.01) and a value of ratio of principal curvatures greater than 5.

Once the candidate keypoints have been detected, the next step is to perform a detailed fit to the nearby data for location, scale, and ratio of principal curvatures [29]. Any points, which have low contrast (and are therefore sensitive to noise) and poorly localized along an edge, should be rejected. In 2001, Lowe [30] adopted a 3D quadratic function to fit local sample points to determine the interpolated of the maximum. The threshold on minimum contrast and threshold on ratio of principal curvatures are applied, the former is to exclude low contrast points and the latter is to remove edge points. Therefore, SIFT provides a set of distinctive points which are invariant to scale, rotation, and translation as well as robust to illumination changes and limited changes of viewpoint [31].  Figure 3 shows the stages of keypoint selection on annular iris image using SIFT.

### 2.3. Orientation Assignment

After determining the keypoints based on SIFT, a main orientation is assigned to each keypoint based on local image gradients. For each image sample *L*(*x*, *y*), the gradient magnitude *m*(*x*, *y*) and orientation *θ*(*x*, *y*) are computed as (3) and (4), respectively,

(3) m(x,y)=(L(x+1,y)−L(x−1,y))2+(L(x,y+1)−L(x,y−1))2,

(4) $$\begin{array}{c} \theta \left(x,y\right)=\text{ta}{\text{n}}^{-1}\left(\frac{L\left(x,y+1\right)-L\left(x,y-1\right)}{L\left(x+1,y\right)-L\left(x-1,y\right)}\right). \end{array}$$

An orientation histogram is formed from the gradient orientations of each keypoint around a certain region. The orientation histogram has 36 bins for 360 degree range; keypoint is weighted by its gradient magnitude and by a Gaussian-weighted circular window with *σ* of 1.5 times of the scale of keypoint before adding it to orientation histogram.

**Figure 4:**
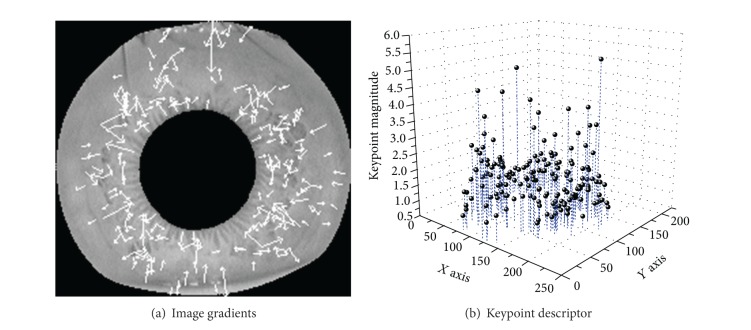
(a) Scale and direction of orientation is indicated by arrow in white color; (b) 3D magnitude representation of detected keypoints from annular iris area.

The highest orientation histogram peaks and any other peaks with amplitudes within 80% of the highest peak are used to create keypoint with the computed orientation. The direction and scale of orientation are indicated by white color as shown in Figure 4.

### 2.4. Keypoint Descriptor Representation

**Figure 5:**
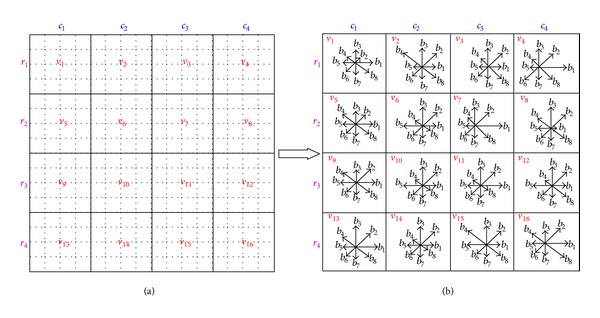
The process of computation of keypoint descriptor. The corresponding gradient magnitude and orientation at each sample point in a region around keypoint location are computed firstly, as shown in (a). Then these samples are accumulated into orientation histograms summarizing the contents over 4 × 4 subregions, with the length of each arrow corresponding to the sum of the gradient magnitudes near that direction within the region, as shown in (b). (a) Image gradients; (b) keypoint descriptor.

In this step, a distinctive descriptor is computed for the local image region. Keypoints are transformed into representation called keypoint descriptors containing the values of all the orientation histogram entries [25]. A keypoint descriptor is characterized by the gradient magnitude and orientation at each keypoint in a region around a keypoint location.  Figure 5 shows the process of keypoint descriptor formed.

Lowe [29] pointed out that 4 × 4 array of histograms with 8 orientation bins for each keypoint achieved the best results; hence, there are 4 × 4 × 8 = 128 element feature vectors for each keypoint. In our work, we also adopt 128 element feature vectors for each keypoint.

## 3. Discriminative Feature Selections

In general, the iris recognition system should select compact and effective features based on the distinct characteristics of the representation data. From the previous work and discussions, the SIFT features may contain redundant features. Therefore, this paper adopts feature selection techniques for suitable feature subset and only select feature with more discriminative information and discard feature of the least useful information. We select discriminative feature based on three strategies, detailed process as follows:

1. to sort orientation probability distribution function (OPDF) based on keypoint's key orientation in descending way and delete corresponding keypoints which have small orientation probability distribution, this operation means reducing number of keypoints;
2. to sort magnitude probability distribution function (MPDF) based on keypoint's neighborhood magnitude in ascending way and delete keypoints corresponding feature elements which have larger magnitude probability distribution, the purpose of this operation is to reduce dimension of feature elements;
3. to reduce number of keypoints and dimension of feature element based on combined above two methods.

The ultimate purpose of select discriminative feature is to realize two goals.

1. minimization of the number of features,
2. maximization of the correct recognition rate and minimization of the equal error rate.

### 3.1. Discriminative Feature Selection Based on Orientation

**Figure 6:**
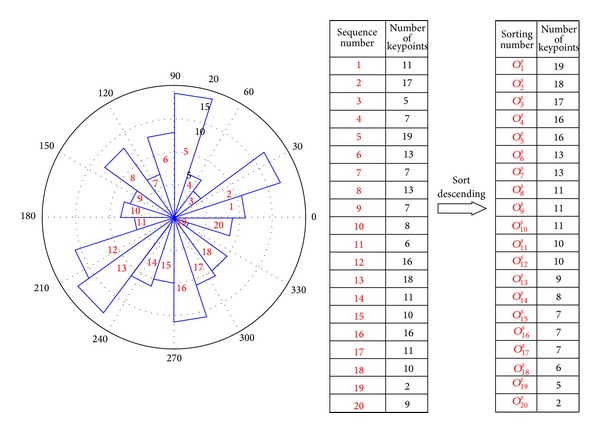
OPDF of keypoints' primary orientation.

OPDF of detected keypoints is shown in Figure 6; from this figure, the key orientation of 216 keypoints shows non-uniform distribution. Here, we equally divide 2*π* into 20 intervals in anticlockwise way, and size of each interval is *π*/10; for convenience, these intervals are denoted from 1 to 20 (tabbed in red color). From Figure 6, it is can be seen 19 keypoints in [2*π*/5, *π*/2]; however, there are only 2 keypoints in [11*π*/5, 23*π*/10].

**Figure 7:**
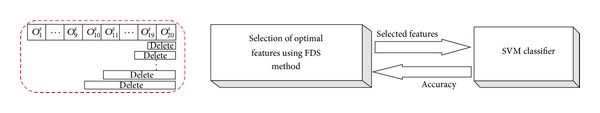
Feature selection process.

We deploy a sorting procedure among the OPDF according to their corresponding keypoint number. The OPDF is denoted by vector **O**, and **O** = [*o*_1_, *o*_2_,…, *o*_20_]. The elements of **O** are sorted in descending way, and denote the sorted vector by *O* ^*s*^; therefore, *O* ^*s*^ = [*o*_1_^*s*^, *o*_2_^*s*^,…, *o*_20_^*s*^]. Further, *O*_*i*_^*s*^ indicates that we only keep the first *i* elements of sorted vector *O* ^*s*^, and *O*_*i*_^*s*^ = [*o*_1_^*s*^, ⋯, *o*_*i*_^*s*^], *i* = 1, ⋯, 20. Owing to images matching method more focuses on key orientated keypoint, therefore, this study utilizes finite delete last several elements corresponding keypoint of *O* ^*s*^, and just keypoints which have larger probability distribution were used as discriminative subfeature. In order to achieve optimal delete scheme, a finite-delete-sorting (FDS) method is used to select the optimal features. For each feature subset, evaluation of discriminative feature requires training the corresponding support vector machine (SVM) and computing its accuracy. The performance of the SVM classifier is estimated using a validation iris image database and is used to guide the FDS as shown in Figure 7.

SVM is a well-accepted approach for pattern classification due to its attractive features and promising performance [32]. For more details about SVM, one can refer to [32], which provides a complete description of the SVM theory. In order to make the linear learning machine work well in non-linear cases, the original input space is mapped into some higher-dimensional feature space by using a kernel function. In this study, radial basis function (RBF) is used as kernel function.

Here, it should be pointed out that the OPDF cannot be used with standard SVM, but the corresponding PDF of feature descriptor (FDPDF) is used as identified feature of SVM. There are two reasons for why we do so. First, the dimension of OPDF vector of each image class is too small (less than 20-dimension) to achieve a satisfactory classification accuracy of SVM necessarily. Second, due to subsequent matching method also based on keypoints descriptor, thus FDPDF is utilized as classification feature. The FDPDF data is normalized by scaling them into the interval of [0, 1] and the classes labels are assigned to FDPDF data. In order to gain an unbiased estimate of the generalization accuracy, the *K*-fold (*K* is set at 10 in this study) cross-validation method is used to evaluate the classification accuracy. In the following experiments, the two important parameters (*C* and *γ*) of SVM are also tuned for three experimental iris image databases.

A ranking procedure among the FDPDF is deployed according to their corresponding classification accuracy rate (CAR) scored by SVM. Instead of using all the keypoints' feature, only the most discriminating FDPDF which own *Max*⁡(CAR(*O*_*i*_^*s*^)) is used as optimal subfeature. For example, assuming that the optimal subfeature is *O*_18_^*s*^ when selecting discriminative feature for Figure 6(a), this result indicates that 7(2 + 5) keypoints are deleted; hence, the numbers of keypoints decrease from 216 to 209.

### 3.2. Discriminative Feature Selection Based on Magnitude

**Figure 8:**
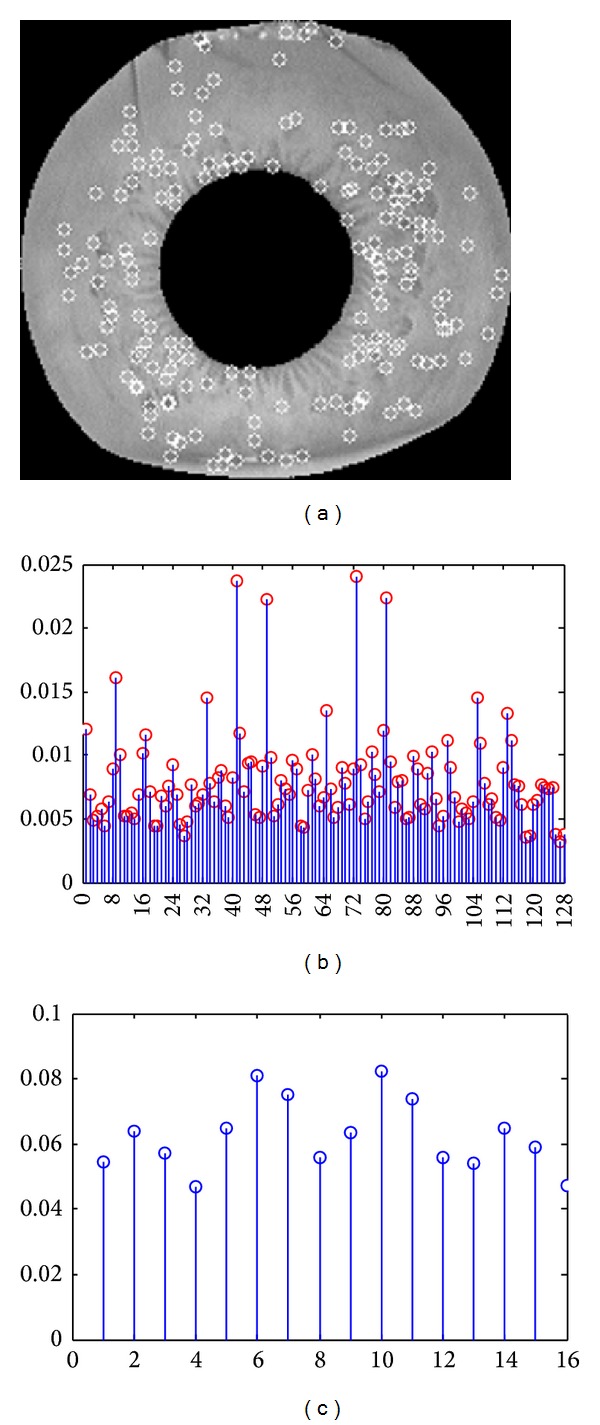
PDF of keypoints' feature descriptor. (a) The detected keypoints, (b) FDPDF (c) NEPDF.

In last subsection, the process of discriminative keypoints selection was introduced. In this section, the process of selecting feature descriptor based on magnitude will be described in detail. We adopt two ways to describe keypoints' descriptor. The first way is FDPDF, which is generated according to the value of feature elements. The second way is neighborhood element probability distribution function (NEPDF), which is generated by 16 neighborhood of a keypoint.  Figure 8 shows the detected keypoints and its corresponding FDPDF and NEPDF.

By comparing Figures 8(b) and 8(c), we can see FDPDF in mixed and disorderly way, but NEPDF in more clear way. Meanwhile, according to previous discussions, a detected keypoint's feature descriptor is generated according to its 16 subregion's 8 accumulated orientation bins magnitude, therefore, we focus on selecting discriminative subfeature based on NEPDF.

The specific processes of FDPDF and NEPDE calculation are as follows. Assuming that an image has *M* keypoints after feature extraction based on SIFT method, and every keypoint with 128-dimension feature elements, then, a matrix **S** of an image with *M* rows and 128 columns can be formed. Let *S*_*α*,*β*_ denote the element in the *α*'th row and the *β*'th in the matrix **S**. A vector *u* ^*α*^, which is called the *α*'th keypoint, can be obtained from the *α*'th of **S** as

(5) uα=[υα,1,υα,2,…,υα,15,υα,16]=[∑i=18Sα,i,∑i=916Sα,i,…,∑i=113120Sα,i,∑i=121128Sα,i].

Further assuming that sum of neighborhood subregion is denoted by *v*_*j*_, then *v*_*j*_ can be computed via

(6) $$\begin{array}{c} v_{j}=\sum_{i=1}^{M}\upsilon_{i,j},\;j=1,\ldots ,16. \end{array}$$

Therefore, the NEPDF *P*(*v*_*j*_) can be calculated through

(7) P(vj)=∑i=1Mυi,j∑i=1M∑j=116υi,j=∑i=1M∑m=8×(j−1)8×jSi,m∑i=1M∑k=1128Si,k,    j=1,…,16.

Further, the FDPDF *P*(*e*_*j*_) can be computed via (8)

(8) $$\begin{array}{c} P\left(e_{j}\right)=\frac{\sum_{i=1}^{M}S_{i,j}}{\sum_{i=1}^{M}\sum_{j=1}^{16}v_{i,j}}=\frac{\sum_{i=1}^{M}S_{i,j}}{\sum_{i=1}^{M}\sum_{k=1}^{128}S_{i,k}},\;j=1,\ldots ,128. \end{array}$$

The NEPDF *P*(*v*_*j*_) is sorted in ascending way, and then also utilize FDS strategy to delete some keypoint descriptor element. The distribution of NEPDF is denoted by vector **V**, and **V** = [*v*_1_, *v*_2_,…, *v*_16_]. The elements of vector **V** are sorted in ascending way, and denote sorted vector by *V* ^*s*^, and *V* ^*s*^ = [*v*_1_^*s*^, *v*_2_^*s*^,…, *v*_16_^*s*^], *V*_*i*_^*s*^ indicates that only keep the first *i* elements of sorted vector, and *V*_*i*_^*s*^ = [*v*_1_^*s*^,…, *v*_*i*_^*s*^], *i* = 1,…, 16. Because standard SIFT adopts Euclidian distance to matching image pairs; therefore, in order to lessen matching distance, this study will finitely delete last several elements of *V* ^*s*^. Here, SVM also is adopted to generate matching accuracy to evaluate the discriminative effectiveness of each feature subset.

**Figure 9:**
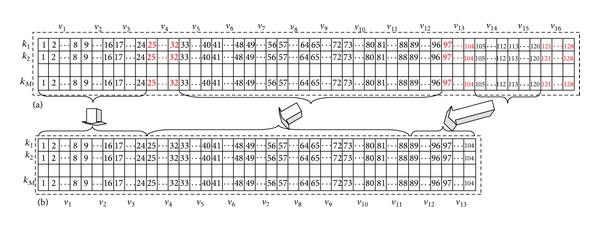
Illustration of feature elements selection based on magnitude. (a) Original feature matrix. (b) Discriminative feature matrix after feature selection based on neighborhood magnitude.

For example, when we select discriminative feature for Figure 8(a) based on magnitude, as shown in Figure 8(c), its sorted vector is [10, 6, 7, 11, 5, 14, 2, 9, 3, 15, 12, 8, 1, 13, 16, 4]. Assume that the optimal subfeature is *V*_14_^*s*^, and this result means feature elements of neighborhood 4, 16, and 13 are deleted.  Figure 9 shows the change process of SIFT feature; the dimension of every keypoint's feature reduces from 128 to 104.

### 3.3. Compounded Feature Selection Based on Orientation and Magnitude

After described discriminative feature selection based on orientation and magnitude, here, compounded feature selection based on combined orientation and magnitude will be introduced in detail. We name the function of compounded feature selection CFS, and the detailed procedure for the CFS is in Algorithm 1.

After getting optimal subfeature by CFS, the number of detected keypoint will be reduced and the feature element with lower dimensions. Iris recognition based on the optimal subfeature will achieve better performance.

## 4. Iris Image Partition and Subpattern Feature Contributions Analysis

### 4.1. Iris Image Partition

In 2009, Belcher and Du [13] assumed that the relative position of features does not change despite scale, rotation, and dilation variance in iris images, and the features close to the pupil will remain close to the pupil and feature on the right side will never be on the right side of the iris. Three years later, in 2012, Soyel and Demirel [25] proposed grid-based approach to overcome the major drawback of standard SIFT technology. They further drew conclusions that there are three advantages of grid-based approach. The first advantage is that local matching within a grid constrains the SIFT features to match features from nearby areas only. The second advantage is increase of speed in matching since the number of features decrease. The major advantage is that grid-based method allows weighting regions, which assure that higher information carrying regions of the image are associated with higher weight values to be considered more significantly.

**Figure 10:**
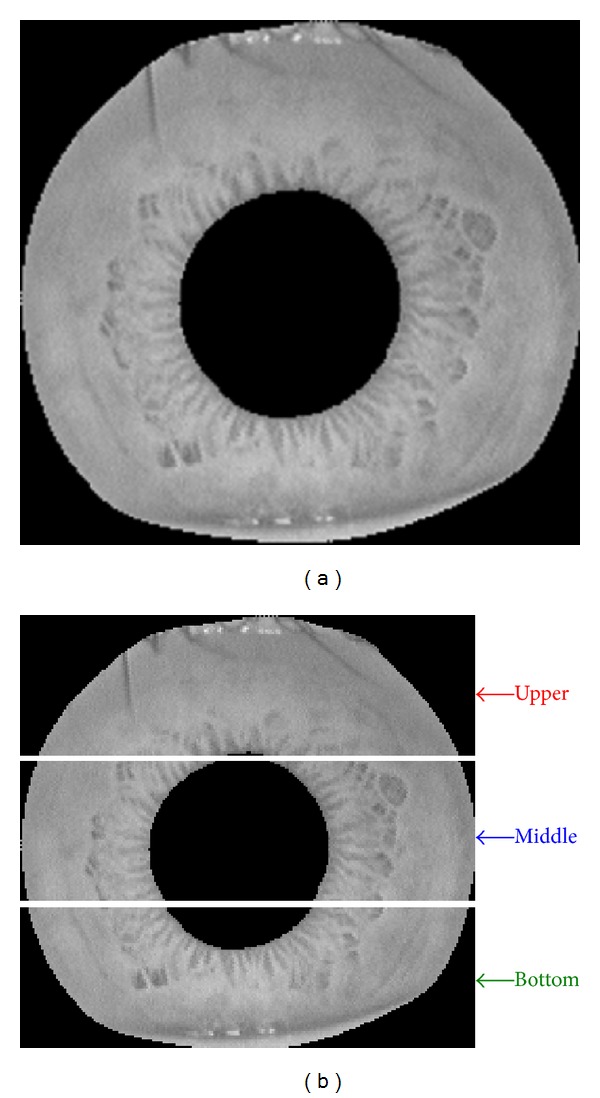
The partition process of segmented iris region. (a) Original segmented iris region. (b) Upper, middle, and bottom subregions.

In subpattern based iris recognition methods, an iris image can be partitioned into a set of equally or unequally sized sub-images depending on user's option. However, how to choose appropriate sub-image size which gives optimal performance is still an open problem [33]; this study will not attempt to deal with this issue in our work. Without loss generality, we divide segmented iris annular area into equally sized partitions in a nonoverlapping way. Segmented annular iris area is divided into three major sub-partition, which are denoted as upper, middle, and bottom sub-regions, and the partition result is shown in Figure 10.

### 4.2. Weights of Subpattern Calculations with PSO

Although papers [13, 25] partition experimental images, these papers did not explain weights assignment process in detail. Moreover, some existing researches have demonstrated that the different segmented iris area has nonuniform feature information. Hollingsworth et al. [34] pointed out that not all the bits in an iris are equally useful. Ma et al. [6] presented that the regions closer to the pupil provide the most useful texture information for recognition. Tsai et al. [35] pointed out that the region closer to pupil usually contains more high-frequency components, the middle region consists of fewer and bigger irregular blocks, and the region closer to limbic is usually covered with the eyelid and sparse patterns. In our previous work, we have proved that different tracks of iris images have different feature information based on local quality evaluation. Therefore, we can draw conclusion that it is unreasonable that upper, middle, and bottom regions have the same weights from above-mentioned analysis. In order to assign reasonable weights for different subregions, this paper adopts training scheme to get related weighted coefficients for corresponding subregions. Meanwhile, particle swarm optimization (PSO) is adopted to accelerate training process. PSO was first developed by Kennedy and Eberhart [36], it is inspired by the social behavior of organisms such as bird flocking and fish schooling, which seeks to explore the search space by a population of particles, and more details can refer to [36].

**Figure 11:**
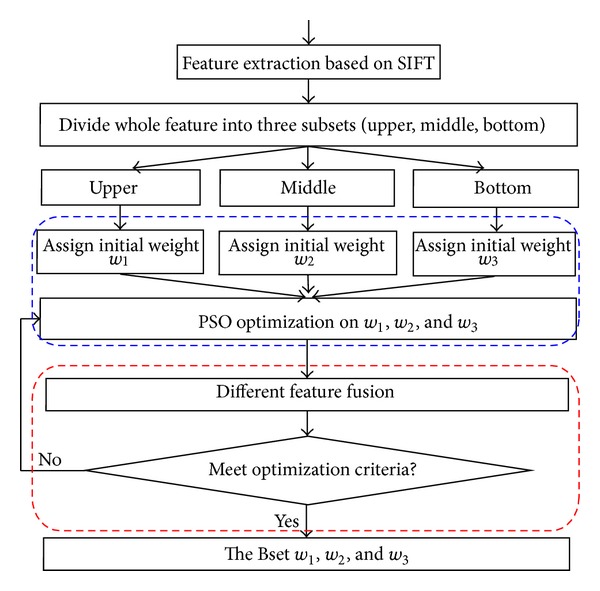
Block diagram for the process of weights assign with PSO.

Assume that *w*_1_, *w*_2_, and *w*_3_ denote the corresponding weights of upper, middle, and bottom subregions, respectively and their values are scaled in the range [0, 1]. Hence, the primary purpose of PSO is to determine the parameters (*w*_1_, *w*_2_, and *w*_3_). To evaluate the improvement of performance achieved by the information fusion, correct recognition rate (CRR) is adopted. CRR indicates the ratio of the number of samples being classified to the total number of test samples correctly. Moreover, two evaluation standards are utilized to assess whether training algorithm to meet end conditions, which are (1) meeting maximum number of iterations and (2) meeting Max(CRR) at many times. In the process of the PSO iterative optimization, the termination condition is the biggest CRR in a certain number of iterations. When meeting (9), then we think the training algorithm meet termination condition

(9) $$\begin{array}{c} \left|\max\left({\text{CRR}}_{i+1}\right)-\max\left({\text{CRR}}_{i}\right)\right|<\varepsilon , \end{array}$$

where *ε* is an extreme minimum value.  Figure 11 shows a block diagram for the process of weights assignment with PSO.

After getting optimal weights for three subregions, without losing generality, these three weights are normalized to Σ*w*_*i*_ = 1.

### 4.3. Weighted Subregion Matching

Matching between two images *I*_1_ and *I*_2_ is performed by comparing each keypoint based on their associated descriptors [28]. Generally, there are four steps in matching process.

**Step 1**

Assume that a keypoint *p*_11_ in *I*_1_ and its closest and second-closest points are *p*_21_ and *p*_22_ in *I*_2_.

**Step 2**

Calculate city distances *d*_1_ = CD(*p*_11_, *p*_21_) and *d*_2_ = CD(*p*_11_, *p*_22_). Here, we should point out that standard SIFT adopts Euclidean distance to compute distance of keypoint pairs; this study presents feature matching solution of using city distance (CD) substitute for Euclidean distance to reduce the computational cost and speed up the matching process. Assuming 2 *n*-dimension vector *x* = (*x*_1_, *x*_2_,…, *x*_*n*_) and *y* = (*y*_1_, *y*_2_,…, *y*_*n*_), the CD of *x* and *y* can be calculated via

(10) $$\begin{array}{c} \text{CD}\left(x,y\right)=\sum_{i=1}^{n}\left|x_{i}-y_{i}\right|. \end{array}$$

**Step 3**

Deciding whether points matching or not. If the ratio *d*_1_/*d*_2_ is smaller than predefined threshold value, then the points *p*_11_ and *p*_21_ are considered to be matching point-pairs. In this paper, a threshold of 0.85 is chosen for the ratio *d*_1_/*d*_2_.

**Step 4**

Decide the matching score between two images based on the number of matched points.

**Figure 12:**
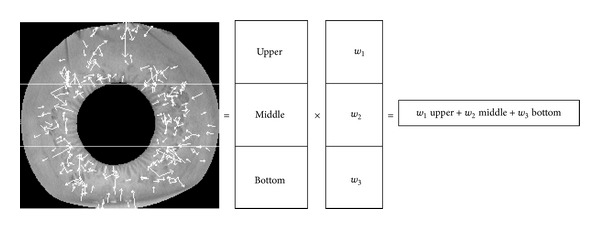
The process of weighted matching.

As shown in Figure 12, such an aggregation is performed through a linear combination of the objectives. In the following experiments, training methods for three iris image databases are adopted to get weighted coefficients.

## 5. Experimental Results and Discussions

### 5.1. Description of Iris Image Databases

**Figure 13:**
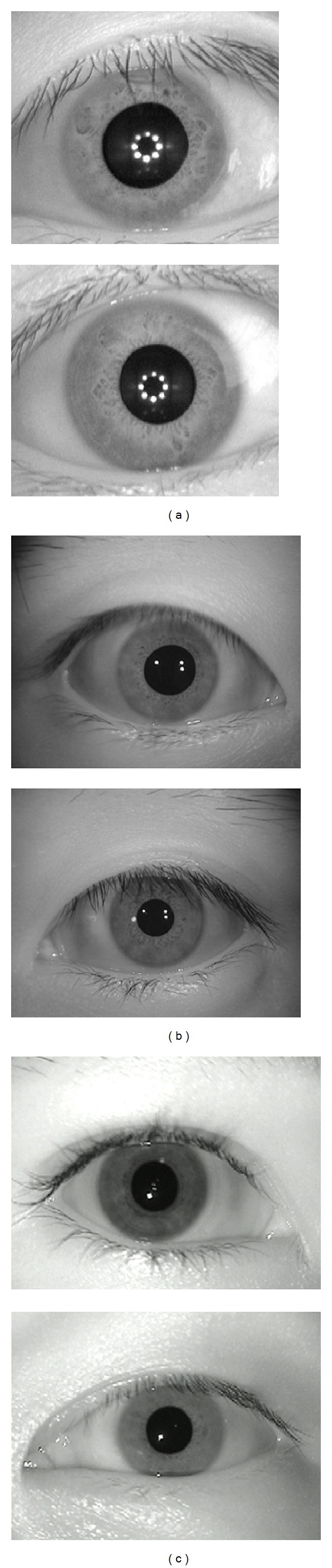
Sample images from CASIA-V3 Interval, CASIA-V3 Lamp, and MMU-V1 iris databases. (a) CASIA-V3-Interval, (b) CASIA-V3 Lamp, and (c) MMU-V1.

Public and free iris image database includes CASIA (four versions) [37]. CASIA database contains near infrared images and is by far the most widely used on iris biometric experiments. The The CASIA-V3 Interval database, which contains 2639 iris images from 395 different classes of 249 subjects, each iris image in this database is an 8-bit gray-level JPEG file with a resolution of 320 × 280 pixels. The CASIA-V3 Lamp was collected using OKI's hand-held iris sensor, which contains 16212 iris images from 819 different classes of 411 subjects. Each iris image in this database is an 8-bit gray-gray JPEG file with a resolution of 640 × 480 pixels. Unlike CASIA-V3 Interval, CASIA-V3 Lamp images are with nonlinear deformation due to variations of visible illumination. MMU-V1 [38] iris database contributes a total number of 450 iris images which were taken using LG IrisAccess2000, these iris images are contributed by 100 volunteers with different age and nationality. They come from Asia, Middle East, Africa, and Europe; each of them contributes 5 iris images for each eye. The iris image databases together form diverse iris representations in terms of sex and ethnicity and conditions under which iris information was captured [39]. In our experiments, 700 iris images from 100 classes are selected randomly on CASIA-V3 Interval, 1000 iris images from 50 classes are selected randomly on CASIA-V3 Lamp, and all 450 images from 90 on MMU-V1 are selected to evaluate the proposed methods. The profiles of the databases used are represented in Table 1. Sample iris images are shown in Figure 13.

### 5.2. Evaluation Protocol

The metrics used for the quantitative evaluation of the proposed algorithm are the following.

1. False accept rate (FAR): the FAR is the probability of accepting an imposter as an authorized subject.
2. False reject rate (FRR): FRR is the probability of an authorized subject being rejected incorrectly.
3. Receiver operating characteristic (ROC) curve: the values of FAR and FRR at various threshold value can be plotted using ROC curve, and ROC curve is used to report the performance of the proposed method.
4. Equal error rate (ERR): the point is in the curve where FAR = FRR is known as ERR [12], and the lower the ERR is, the better the algorithm is.
5. Correct recognition rate (CRR): several images are tested with one-to-many matching. The CRR is the ratio of the number of images correctly classified to the total number of tested images.

### 5.3. Experimental Methodology

To measure the performance of the proposed algorithms, extensive experiments are carried out at various levels. Here, we mainly focus on six major sets experiments.

(1) The first set of experiments aim at selecting the optimal subfeature based on orientation. To achieve this purpose, the normalized FDPDF data of *O*_*i*_^*s*^ (*i* = 10,11,…, 20) are divided into 10 subsets, respectively. Each time, one of the 10 subsets is used as the test set and the other 9 subsets are put together to form a training set. Then the average error across all 9 trials is computed. Finally, we design two loops of cross-validation [40, 41] to tune two important parameters (*C* and *γ*) of SVM for three experimental iris image databases. The inner loop is used to determine the optimal parameters of the SVM classifier and the outer loop is used for estimating the performance of the SVM classifier. The parameter *C* is set at 512, 8, and 2 and *γ* at 0.03125, 0.125, and 0.65 for the CASIA-V3 Interval, CASIA-V3 Lamp and MMU-V1, respectively, when the highest classification accuracy has been achieved with the RBF kernel.

(2) The purpose of the second set of experiments is to select the optimal subfeature based on magnitude. In order to gain an unbiased estimate of the generalization accuracy, the 10-fold cross-validation method is used to evaluate the classification accuracy of the normalized NEPDF data of *V*_*i*_^*s*^ (*i* = 8,9,…, 16) by SVM classifier. The parameter *C* is set at 64, 16, and 32 and *γ* at 0.064, 0.25, and 0.10 for the CASIA-V3 Interval, CASIA-V3 Lamp, and MMU-V1, respectively.

(3) The goal of the third set of experiments is to further get optimal subfeature based on compounded feature selection strategy combined orientation and magnitude. Assuming that *C*_*i*_^*j*^ (*i* = 15,…, 20, *j* = 12,…, 16) denotes the compounded subfeature, subscript *i* correspond to *O*_*i*_^*s*^ and superscript *j* correspond to *V*_*j*_^*s*^. Similar to the second set of experiments, the normalization NFPDF data of *C*_*i*_^*j*^ as input to SVM classifier which also adopt the 10-fold cross-validation strategy. The parameter *C* is set at 256, 32, and 16 and *γ* at 0.25, 0.63, and 0.018 for the CASIA-V3 Interval, CASIA-V3 Lamp, and MMU-V1, respectively.

(4) The fourth set of experiments is to analyze the performance of three proposed subfeature selection strategies. The performance is evaluated in verification mode and using ROC curve, which include EER, CRR, FAR, and FRR evaluation protocols.

(5) In order to evaluate the performance of the proposed weighted matching method, the fifth set of experiments are as follows. Firstly, we get the matching rates of bottom, middle, and upper three subregions of annular segmented iris, respectively. Secondly, we assign corresponding weighted coefficients for three subregions. Finally, we get whole recognition accuracy rate and equal error rate.

(6) In order to further analyze the efficiency of our proposed methods, we carry out some quantitative comparisons with some existing state-of-the-art methods.

All approaches are implemented using C++ (with OpenCV) and MATLAB 9.2 platform and simulated on 2.53 GHz Intel Core i3 CPU with 2.0 GB RAM.

### 5.4. Experimental Results and Performance Evaluation

In this section, we focus on analyzing the performance of our proposed methods which include discriminative feature selection strategies and weighted matching approach.

**Figure 14:**
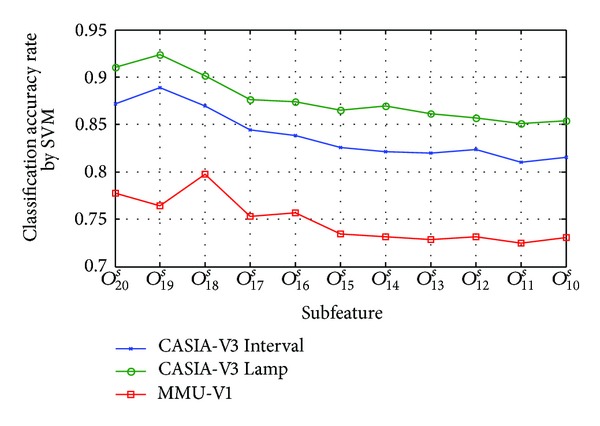
Classification accuracy of subfeature based on orientation selection by SVM classifier.

Figure 14 shows the classification accuracy of subfeature based on orientation selection strategy by SVM classifier; *O*_20_^*s*^ means the all detected keypoints' features. From this figure, an overall drop trend of classification accuracy rate (CAR) is observed from subfeature *O*_19_^*s*^ to subfeature *O*_10_^*s*^ on CASIA-V3 (Interval and Lamp) iris databases, and the CAR of *O*_19_^*s*^ exhibits the highest CAR of 88.86% for CASIA-V3 Interval and 92.35% for CASIA-V3 Lamp, respectively; these experimental results show that the *O*_19_^*s*^ is the optimal subfeature for CASIA-V3 database. Similarly, the highest CAR of 79.78% is achieved in subfeature *O*_18_^*s*^ for MMU-V1, which further shows that *O*_18_^*s*^ is the optimal subfeature for MMU-V1 databases. Experimental results mean that subfeature selection achieved ideal effect.

**Figure 15:**
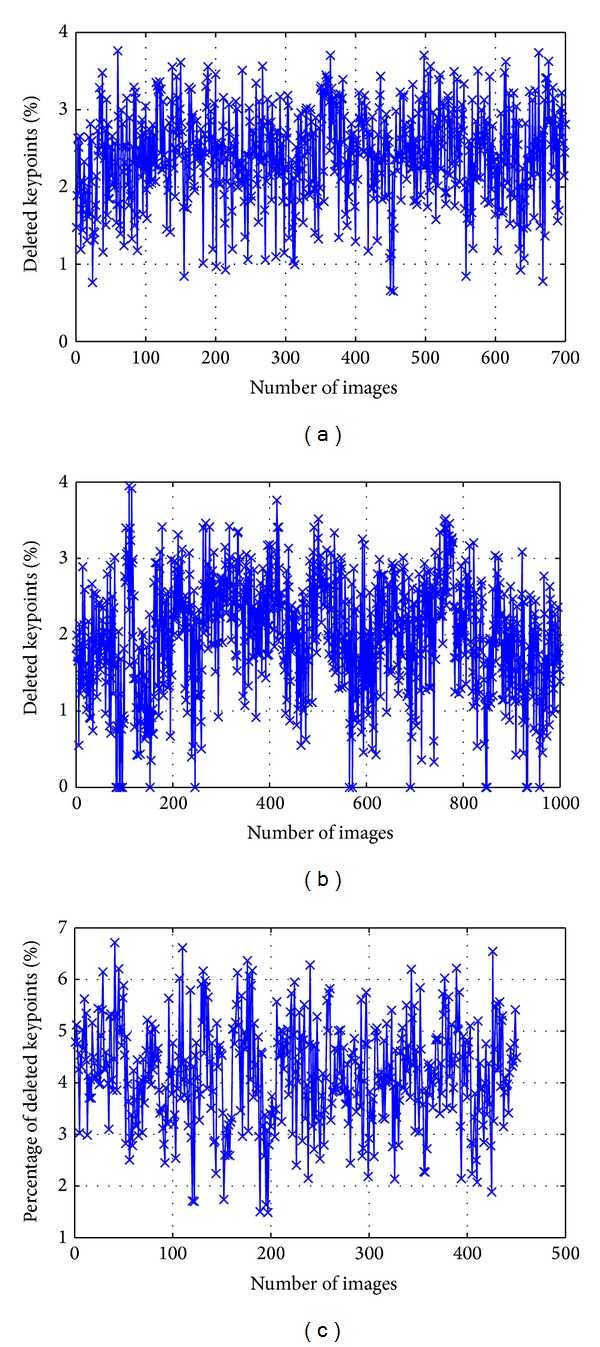
Percentage of deleted keypoints based on orientation. (a) CASIA-V3 Interval, (b) CASIA-V3 Lamp, and (c) MMU-V1.

The diagrams of percentage of deleted keypoints based on orientation are shown in Figure 15. From this figure, it can be seen that the maximum percentages of deleted keypoints for single iris image are 3.76%, 3.95%, and 6.71% for CASIA-V3 Interval, CASIA-V3 Lamp, and MMU-V1, respectively.

**Figure 16:**
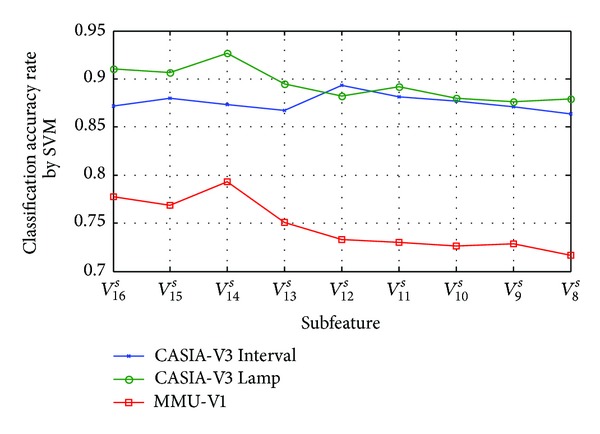
Classification accuracy of subfeature based on magnitude selection by SVM classifier.

Figure 16 shows the classification accuracy of subfeature based on magnitude selection strategy by SVM classifier. In this figure, subfeatures *V*_12_^*s*^, *V*_14_^*s*^, and *V*_14_^*s*^ achieve the highest CAR for CASIA-V3 Interval, CASIA-V3 Lamp, and MMU-V1 database, respectively. For CASIA-V3 Interval, the CAR of all feature elements is 87.16%, but the CAR of *V*_12_^*s*^ is 89.29%. The CAR of *V*_14_^*s*^ is 92.70%, which is higher than that of *V*_16_^*s*^ (91.02%) for CASIA-V3 Lamp. In MMU-V1, its highest CAR is 79.28%, which is beyond absolute value 1.50% compared to CAR of *V*_16_^*s*^. Hence, the subfeatures *V*_12_^*s*^, *V*_14_^*s*^, and *V*_14_^*s*^ are thought as the optimal subfeatures for CASIA-V3 Interval, CASIA-V3 Lamp, and MMU-V1, respectively.

**Figure 17:**
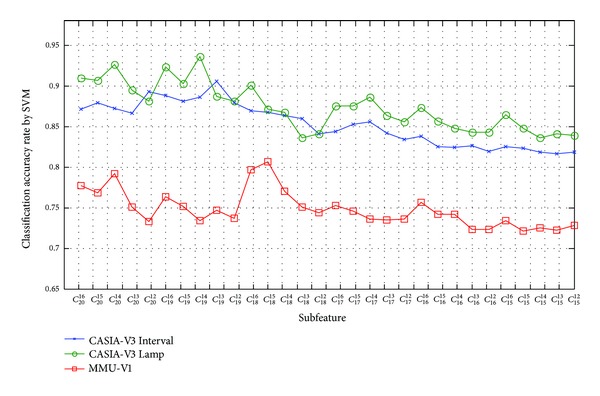
Classification accuracy of subfeature based on compounded selection by SVM classifier.

Figure 17 shows the classification accuracy of subfeature based on compounded selection strategy by SVM classifier. From this figure, it can be seen that the highest classification accuracy of 90.65%, 93.68%, and 80.68% on *C*_19_^13^, *C*_19_^14^, and *C*_18_^15^ for CASIA-V3 Interval, CASIA-V3 Lamps and MMU-V1 database, respectively. Therefore, the subfeatures *C*_19_^13^, *C*_19_^14^ and *C*_18_^15^ are thought as the optimal subfeatures for CASIA-V3 Interval, CASIA-V3 Lamps and MMU-V1 based on compounded feature selection strategy.

Considering Figures 15, 16, and 17 as a whole, we can seen that *C*_19_^13^ achieves the highest CAR compared to *O*_19_^*s*^ and *V*_12_^*s*^ for CASIA-V3 Interval, and *C*_19_^14^ achieves the highest CAR for CASIA-V3 Lamp, and the CAR of *C*_18_^15^ is the highest CAR for MMU-V1. Therefore, we can safely conclude that compounded selection strategy is the best subfeature selection method of all the three proposed feature selection methods.

Table 2 shows the different subregions' weighted coefficients assignment for three iris image databases; the experimental results further demonstrate that it is unreasonable to simply assign the same weights to three subregions.

**Figure 18:**
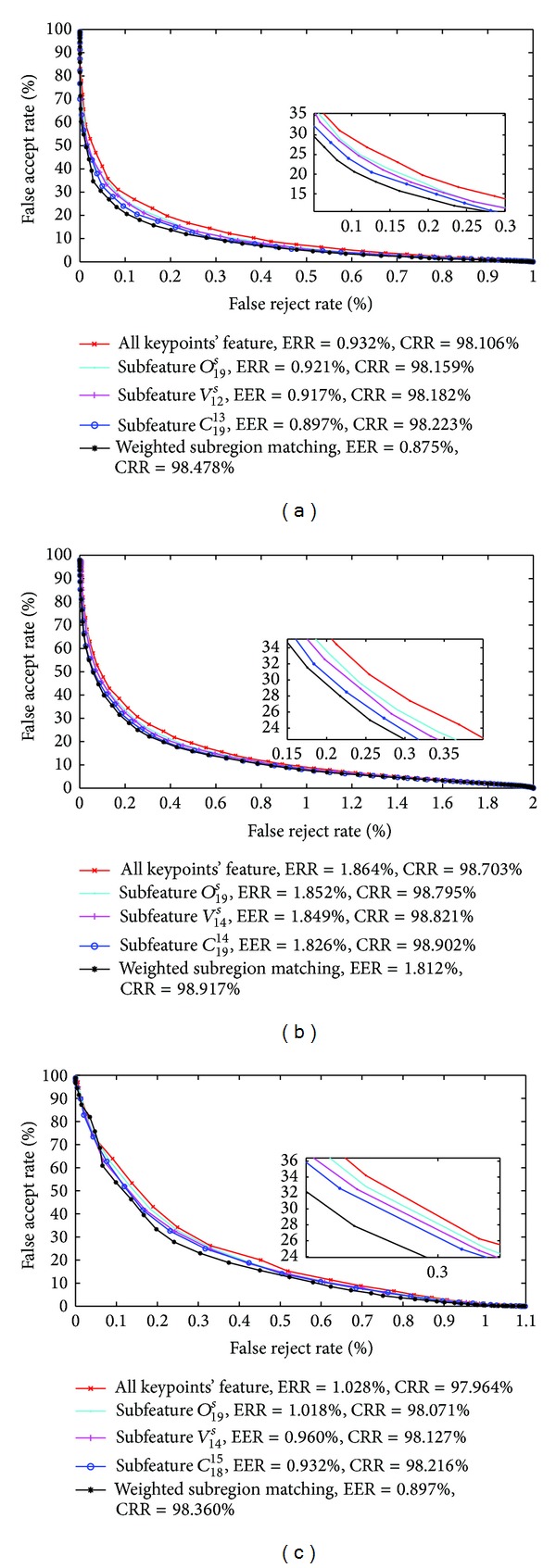
ROC curves of all keypoints feature and three subfeature selection strategies and weighted matching method. (a) ROC curve for CASIA-V3 Interval, (b) ROC curve for CASIA-V3 Lamp, and (c) ROC curve for MMU-V1.

In order to further evaluate the performance of subfeature selection strategies and weighted matching method, only the optimal subfeature is used for comparing to the original whole feature in verification mode. Hence, we get FAR, FRR, and EER values for CASIA-V3 Interval, CASIA-V3 Lamp, and MMU-V1 databases.  Figure 18 shows the ROC curve of FAR/FRR for three iris image databases, respectively.

From Figure 18, we find that subfeature has less EER than the all keypoints' feature, and it is observed that the EERs are 0.932%, 1.864%, and 1.028% for all keypoint's feature of CASIA-V3 Interval, CASIA-V3 Lamp, and MMU-V1, respectively. After adopting subfeature strategy based on orientation, its corresponding ERRs values are decrease to 0.921%, 1.852%, and 1.018% and decreasing to 0.917%, 1.849% and 0.960% after subfeature based on magnitude; they further reduce to 0.897%, 1.826%, and 0.932% after adopting compounded subfeature strategy, respectively.  Figure 18 also demonstrates a comparison of CRR at EER for subfeature and whole feature. From the comparison results, it is evident that the CRRs of subfeatures which are selected by feature selection strategies are high compared to CRRs of whole feature for three databases.

When further analyze the experimental results, we can find that weighted subregion matching performs reasonably well in term of EER and CRR for all of the three databases. EERs of weighted subregion matching are 0.875%, 1.812%, and 0.897% for CASIA-V3 Interval, CASIA-V3 Lamps and MMU-V1 database, respectively, which are the best results in all of EERs. CRRs of weighted subregion matching also achieved encouraging results if we take into account 98.478%, 98.917%, and 98.360% for CASIA-V3 Interval, CASIA-V3 Lamps and MMU-V1 databases.

**Figure 19:**
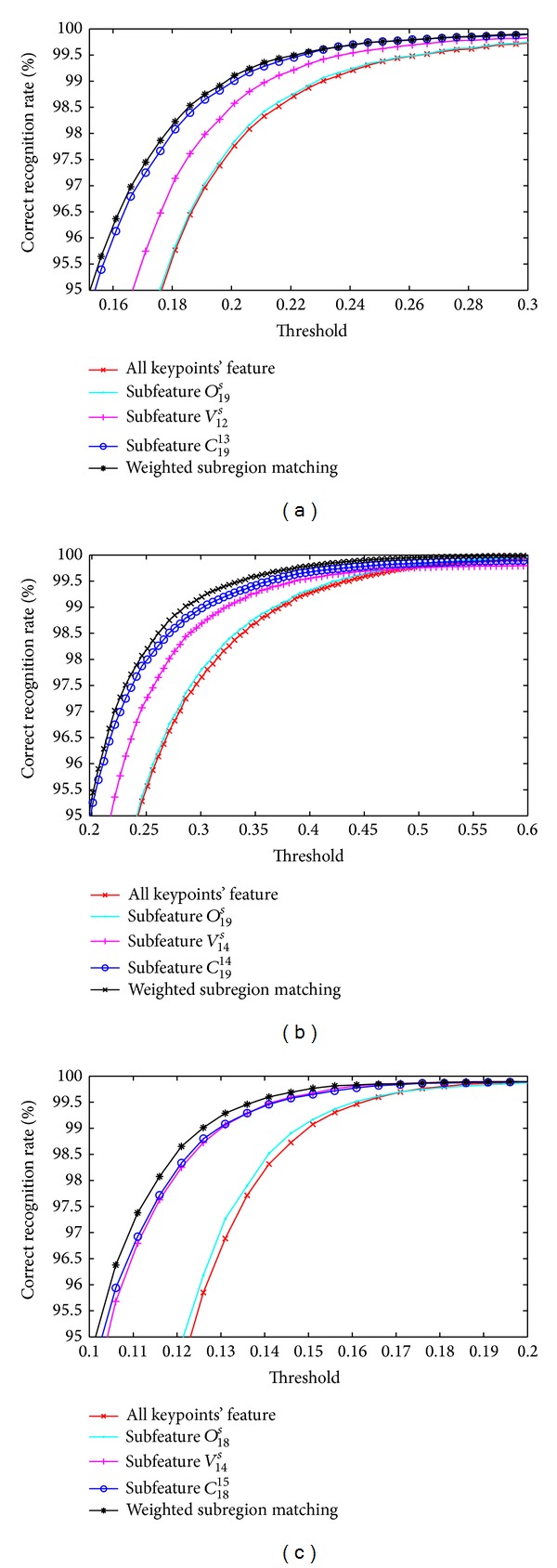
CRR curve of proposed methods under different threshold. (a) CRR curve for CASIA-V3 Interval, (b) CRR curve for CASIA-V3 Lamp, and (c) CRR curve for MMU-V1.

Figure 19 shows the CRR curve of our proposed methods under different thresholds; here, it should be pointed out that the threshold is to judge whether two images are matching or not by utilizing the ratio of matching point pairs. From Figure 19, it is observed that with the increase of thresholds, the CRRs also increase rapidly for three databases. Moreover, it is still observed that the highest CRRs are achieved by weighted subregion matching method, and the highest CRRs are 99.82%, 99.93%, and 99.75% for CASIA-V3 Interval, CASIA-V3 Lamp, and MMU-V1 databases, respectively.

From above experimental results, it is can be safely concluded that our proposed methods, which include subfeature selection strategies and weighted subregion matching approaches, are effective methods and can achieve low EER and high CRR.

### 5.5. Comparison with Existing Methods

In this stage, in order to further exhibit the efficiency of our proposed approach, we carry out a series of experiments to provide a comparative analysis of our proposed method with some state-of-the-art methods in terms of CRR and EER based on CASIA-V3 databases. As described in introduction section, there are five feature extraction and recognition approaches in existing literatures. The comparative works shown in Table 3, which are the best known among existing schemes iris recognition, can be divided into these five categories. Therefore, comparison results further demonstrate encouraging performance of our proposed methods. Table 3 summarizes the best results obtained by each method.

We would like to point out here that in order to achieve unbiased comparisons results, some experimental results directly come from some published work, which are carried out on CASIA-V3 Interval and Lamp databases and shown in Table 3. From this table, it is observed that method reported in [42] has better CRR than the proposed method on CASIA-V3 databases. Bouraoui et al. [42] got those result by giving the accuracy that is defined as equal to 100 − (FAR + FRR)/2; however, this hypothesis may be unreasonable for performance evaluation. From Table 3, we can see that our proposed methods have less EER than the methods reported in [1, 6, 7, 9, 13, 42] preceded by the method proposed in [8, 43, 44] on the CASIA-V3 Interval and find that EER achieved in papers [4, 11, 45, 48] has less than our methods but EER achieved in papers [11, 45] has higher than that of the proposed methods this study on the CASIA-V3 Lamp database.

We also provide the computation complexity comparison between the various known methods and the proposed methods. From Table 4, it is observed that our proposed methods consume less time than the other methods reported in Table 4 if we take into account the whole time consumption. Here, it should be pointed out that the experimental results on CASIA-V1 database reported in Ma et al. [7] were achieved in a machine of 128 M RAM running at 500 MHz speed. Our experimental environment is better than Ma's experimental environment; nevertheless, the resolution of CASIA-V1 image is 320 × 280 pixels, which is equal to the resolution of CASIA-V3 Interval image but less than the resolution of CASIA-V3 Lamp image (640 × 480 pixels). The proposed method is still computationally effective if taking into consideration processing high resolution image consume more time. The experimental results achieved in [47] were conducted on at 3.00 GHz Pentium IV PC with 1 GB RAM; this experimental environment is similar to ours and comparison results also demonstrate that our proposed methods' computation complexity is still less compared to [47].

In 2008, Roy and Bhattacharya [47] pointed out that feature subset selection algorithms can be classified into two categories, which are the filter and wrapper approaches, based on whether the feature selection is performed independently of the learning algorithm or not to construct the verifier; our proposed subfeature selection methods should fall into filter category. Meanwhile, Roy and Bhattacharya [47] further pointed out are the major drawback of filter approach is that subfeature selection may depend on the representational and inductive biases when building the classifier. However, since our proposed subfeature methods are related to keypoints' intrinsic property, which are orientation and neighborhood magnitude of keypoints, the proposed subfeature selection strategies are able to overcome the drawback of filter approach and achieve ideal result.

## 6. Conclusion and Future Work

In this paper, we develop an iris recognition system based on optimal subfeature selection strategies and a weighted subregion matching method. Firstly, we describe the process of feature extraction and feature presentation based on SIFT. Then, we propose three subfeature selection strategies. Finally, weighted subregion matching is proposed to get the whole final matching result. Three public accessible databases, which are (1) CASIA-V3 Interval, (2) CASIA-V3 Lamp, and (3) MMU-V1 databases, are used in a series of experiments. The experimental results and comparison results demonstrate that our proposed methods can effectively improve the performance of iris recognition system with respect to CRR and EER.

From the experimentation, it is observed that our proposed discriminative subfeature selection strategies are able to discard the redundant keypoints and reduce the dimension of corresponding keypoints descriptor representation, and compounded feature selection method achieves the best effect among three proposed optimal feature selection strategies. The proposed subregion matching method can effectively overcome the major drawback of standard SIFT technology unconsidering the location of the feature. Moreover, assign weighted coefficients for three subregions of segmented iris by training scheme accord with iris intrinsic feature distribution characteristics, and PSO method also accelerates training process effectively. Based on getting reasonable weights, weighted subregion fusion strategy is able to further achieve encouraging performance.

More attention will be paid to evaluate the proposed system in more other iris image databases. In addition, we will continuously focus on investigate feature selection strategy and feature fusion method.

## Figures and Tables

**Algorithm 1:**
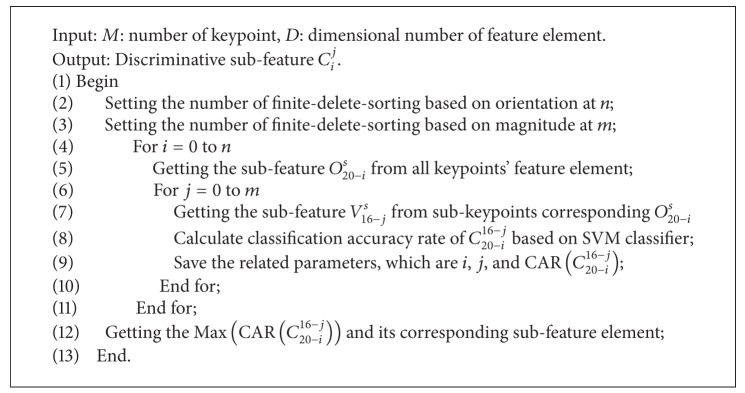
Function CFS.

**Table 1:** The profiles of iris image databases.

Image databases	No. of images	No. of classes	Images per class	Intraclass combinations	Interclass combinations
CASIA-V3 Interval	700	100	7	1200	118,800
CASIA-V3 Lamp	1000	50	20	5000	245,000
MMU-V1	450	90	5	540	48,060

**Table 2:** Weighted coefficients assignment on subregion for three databases.

Iris image databases ↓ weights of subregion of annular iris area →	Upper	Middle	Bottom
CASIA-V3 Interval	0.1000	0.4000	0.5000
CASIA-V3 Lamp	0.2941	0.5294	0.1765
MMU-V1	0.7692	0.0770	0.1538

**Table 3:** Comparisons of CRR and EER.

Methods	Correct recognition rate (%)		Equal error rate (%)	
	CASIA-V3 Interval	CASIA-V3 Lamp	CASIA-V3 Interval	CASIA-V3 Lamp
Bouraoui et al. [42]	99.97	99.97	1.74	18.21
Ma et al. [6]	94.90*	94.82	2.62*	—
Ma et al. [7]	95.54*	96.35	2.07*	—
Daugman [1]	95.19*	96.13	1.80*	3.47^#^
Roy et al. [43]	97.21*	97.86	0.71*	—
Masek and Kovesi [44]	97.61^@^	98.19	0.584^&^	—
Mehrotra et al. [12]	98.55	—	—	—
Belcher and Du [13]	—	—	2.1^&^	—
Tsai et al. [8]	—	—	0.148	—
Zhu et al. [9]	—	—	3.53^&^	—
Sun amd Tan [10]	—	—	—	1.22
Zhang et al. [11]	—	—	—	0.37
Daugman [2]	—	—	—	1.05^*∧*^
Wildes et al. [4]	—	—	—	0.86^*∧*^
He et al. [45]	—	—	—	0.75^*∧*^
Proposed	99.82	99.93	0.78	0.82

Note. Data labeled by *come from Roy et al. [43], ^#^come from Zhang et al. [11], ^@^come from Mehrotra et al. [12], ^&^amp; come from Tsai et al. [8], and ^*∧*^come from He et al. [45].

**Table 4:** Comparison of the computation complexity.

Methods	Feature extraction (ms)	Matching (ms)	Feature extraction + Matching (ms)	Others
Daugman [3]	682.5*	4.3*	686.8*	—
Wildes et al. [4]	210.0*	401.0*	611.0*	Registration
Boles and Boashash [5]	170.3*	11.0*	181.3*	—
Ma et al. [6]	260.2*	8.7*	268.9*	Feature reduction
Ma et al. [7]	244.2*	6.5*	250.7*	—
Roy and Bhattacharya [46]	80.3^*∧*^	167.2^*∧*^	247.5^*∧*^	Feature reduction
Roy and Bhattacharya [47]	20.3^*∧*^	130.4^*∧*^	150.7^*∧*^	Feature reduction
Proposed (CASIA-V3 Interval)	89.6	48.7	138.3	Feature reduction & sub-region fusion
Proposed (CASIA-V3 Lamp)	96.7	53.0	149.7	Feature reduction & sub-region fusion

Note. Data labeled by *come from Ma et al. [7] and ^*∧*^come from Roy and Bhattacharya [47].
